# Why Should Radiologists Evaluate MR Localizer Sequences?

**DOI:** 10.7759/cureus.43667

**Published:** 2023-08-17

**Authors:** Ebru Hasbay

**Affiliations:** 1 Department of Radiology, Tepecik Education and Research Hospital Center, University of Health Sciences, Izmir, TUR

**Keywords:** discopathy, unsuspected, spinal, incidental, magnetic resonance spine localizer

## Abstract

Aim: This study aimed to assess the diagnostic accuracy of magnetic resonance (MR) localizer sequences in the detection of spinal incidental findings.

Materials and methods: MR localizer sequence findings from 384 patients were reviewed retrospectively. The images were evaluated by an experienced radiologist. T2-weighted diagnostic sagittal and coronal images included in the abdominal images were taken as references.

Results: Of the 384 patients, 170 were female and 214 were male. Pathology was detected in 63 of the patients. The findings were more common in male groups. These pathologies were spinal discopathy, metastases, hemangioma, angulation in the coccyx, and hemivertebra.

Conclusions: Although often overlooked, MR localizer images enable diagnosing additional pathologies in the spine. These are unsuspected but can be critical for patient management, reducing patient morbidity and mortality.

## Introduction

Medical imaging is becoming increasingly popular around the world. A recent study found that the number of imaging examinations performed in the United States increased significantly between 1996 and 2010. In this regard, magnetic resonance imaging (MRI) grew faster than previous imaging techniques [1]. MRI is a relatively new imaging technique that was initially used in medicine towards the end of the 1970s, not long after computed tomography (CT) uses [2].

Due to significant constraints on radiology departments in increasing efficiency and throughput, radiologists are under pressure to minimize reporting times. Some of our colleagues shorten reporting times by skipping review localizer sequences, despite their poor quality due to low resolution and saturation bands obscuring the images. Thick slices can also indicate pathology and are occasionally visible only on a single scan. Furthermore, MR imaging assessment is a very complex procedure that is prone to inaccuracy due to a number of cognitive and perceptual biases. They include missed findings, the satisfaction of search or satisfaction of report, and location type error (key finding is missed because it lies outside of the area of interest) which can lead to incidental findings being ignored by the interpreter [3].

Although the vast majority of incidental observations are asymptomatic, they might create ethical and legal concerns, particularly when overlooked and later proven to be clinically relevant. As a result, the radiologist is responsible for reporting these incidental findings and making necessary suggestions. This is likely to be even more important when the findings are outside of the reporting radiologist's or clinician's field of competence [4].

Few researchers have previously explored the reliability of scout CT lateral radiographs (sCT), but despite their potential benefits, no one has considered MR localizer sequences [5-7]. Given the growing number of MRI examinations and the potential of its sequences, it appears that MR localizer validation is required. The purpose of this study was to investigate the reliability of MR localizer sequences obtained for spine assessment in terms of incidental findings.

## Materials and methods

Patient selection

Abdominal MRI examinations of the 384 patients performed between January 2020 and December 2022 were retrospectively and randomly collected from the radiology digital archive of our institution. "Incidental finding" was defined as any abnormal vertebral finding that entered the imaging field. The image recruitment exclusion criteria were confined to (a) patients younger than 18 years old, (b) MRI focused solely on the cervical or thoracic spine, (c) and, of course, the lack or illegibility of MR localizer sequences.

Imaging technique

MRI examinations were performed with the standard protocol using a 1.5 T MRI system (Siemens Avanto, Siemens Aera, GE Optima360; Erlangen, Germany: Siemens Medical Solutions). The protocol included sagittal, axial, and coronal T2-weighted images without fat saturation, axial T2-weighted fat-saturated images, and axial T1-weighted fat-saturated gradient echo images before and after intravenous contrast administration (gadoteric acid, Dotarem, 0.1 mmol/kg).

Statistical analysis

SPSS version 17.0 software (Armonk, NY: IBM Corp.) was used for statistical analysis. Analytical approaches (Kolmogorov-Smirnov/Shapiro-Wilk tests) were used to determine the normal distribution of the variables. For regularly distributed variables, descriptive analyses were performed using the mean and standard deviation. In categorical data, descriptive statistics were conducted by providing frequency and percentage values. To compare paired groups in continuous data, t-tests were used in independent groups with a normal distribution. In the study of categorical data, Pearson's chi-square test was performed. Cases with p-values less than 0.05 were considered statistically significant.

## Results

**Figure 1:**
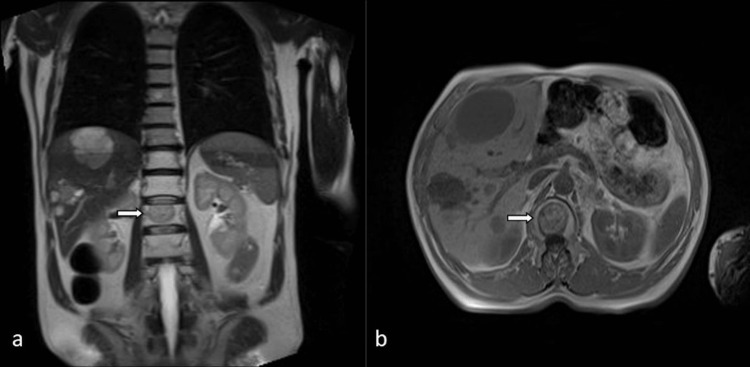
Vertebral metastases in a 61-year-old male patient. MR localizer sequence (a) and axial T1-weighted image (b) show sharply demarcated metastatic lesion (arrows).

**Figure 2:**
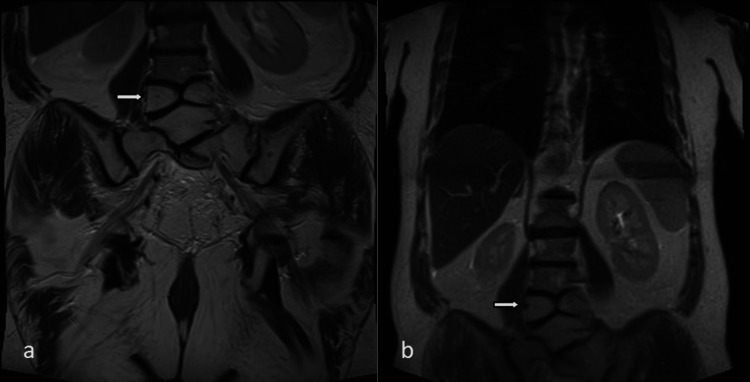
L4 hemivertebra in a 51-year-old male patient. Coronal T2-weighted image (a) and MR localizer sequence (b) show hemivertebra (arrows).

Data from 384 patients were taken into the study. The mean patient's age was 40.9±16.7 ranging from 16 to 77 years. This study's population included 214 male and 170 female patients. When the data was analyzed, 16.4% (n=63) patients had pathology and 83.6% (n=321) patients did not. A total of 66.3% (n=47) of patients had lumbar discopathy and 22.5% (n=16) had thoracic discopathy. Metastasis was found in three patients, coccygeal angulation in one patient, hemivertebra in one patient, and hemangioma was found in two patients (Figures 1a, 1b, 2a, 2b). The findings were more common in male groups. Distributions of the lesions are summarized in Table 1.

**Table 1:** Distribution of the lesions.

Lesions and localizations	Number of cases (%)
Lumbar discopathy	47 (66.3%)
Thoracic discopathy	16 (22.5%)
Lumbar discopathy + thoracic discopathy	1 (1.4%)
Vertebral hemangioma + discopathy	2 (2.8%)
Metastasis + discopathy	3 (4.2%)
Coccygeal angulation + discopathy	1 (1.4%)
Hemivertebra + discopathy	1 (1.4%)

There was no statistically significant difference between general pathology and lumber pathology distributions (p=0.096) (Table 2). However, a statistically significant difference was observed with distributions of lumbar pathology and thoracic pathology groups when compared to general pathology groups (p<0.0001). A statistically significant difference was also found between the distribution of the coccygeal pathology group and other pathology groups (general, lumbar, and thoracic) (p<0.0001).

**Table 2:** Comparison of pathological findings. Pearson's chi-square test was applied and a p-value of <0.05 was accepted as statistically significant.

Groups	Frequency (n)	Percentage (%)	p-Value (chi-squared value)		
			P2	P3	P4
General pathology (P1)	63	16.4	p=0.096 (2.8)	p<0.0001 (31.7)	p<0.0001 (65.3)
Lumber pathology (P2)	47	12.2	-	p<0.0001 (16.9)	p<0.0001 (46.6)
Thoracic pathology (P3)	16	4.1	-	-	p=0.0003 (12.94)
Coccygeal pathology (P4)	1	0.3	-	-	-

## Discussion

A careful examination of such localizer images may aid in the detection of vertebral diseases, such as dischopathy, hemangioma, coccygeal angulation, and even vertebral metastases. In this study, discal pathologies were the most common incidental findings. Most of these coincidental findings are benign [8]. Although the lesions that are thought to be benign are often not emphasized, their effects on human health are not clear [4,9]. Some of these lesions may be the initial stages of some diseases and therefore they should be evaluated systematically.

Vertebral hemangiomas account for up to 4% of all spinal tumors [10]. They are benign vascular lesions of the vertebral body that can often be easily identified via radiological imaging [11]. Although it is a common lesion, less than 1% gives neurological signs [12,13]. In this study, thoracic vertebral hemangioma was detected in one patient.

In this study, one patient had coccygeal angulation, which was anterior angulation. The anatomy of the coccygeal region is complex, which may contribute to or be the cause of coccyx region pain (coccydynia). Coccydynia is a prevalent illness that is known for being difficult to diagnose and cure. Imaging, on the other hand, can help identify potential causes of pain and the right source of management.

Vertebral metastases represent the involvement of the vertebral spine by hematogenously disseminated metastatic cells. Up to 70% of individuals with cancer show spine metastatic disease at autopsy, indicating that the vertebral column is a common location for metastatic illness [14]. As a result, they must be considered and reported in any differential diagnosis of a spinal bone lesion in a patient over the age of 40 years. The current investigation discovered thoracic and lumbar spine metastases in three patients.

In this investigation, one patient was discovered to have multiple levels of hemivertebrae with scoliosis. Hemivertebrae is caused due to the failure of development of one of the chondrification centers of vertebra, resulting in an unpaired sclerotome and hemivertebra [15].

The growing number of patients and MR imaging requests cause radiologists to struggle to keep up, resulting in long appointment wait times for patients and delays in diagnosis and treatment. As a result, radiologists evaluating all sequences and maximizing utility from all available images will lessen the load on both the patient and the imaging units. Localizer sequences are vital in precise localization since they recognize many coincidental diseases. In a study, Bazzocchi et al. evaluated MR localizer sagittal sequences to aid in the diagnosis of spinal fracture [16]. They advocated in their study how simple the MR localizer sequence is and how it should be included in daily practice evaluations due to its contribution to identifying vertebral fractures. There is also a recently published study that emphasizes the importance of magnetic resonance spine localizers [17].

There were some limitations to this investigation. First, the study's retrospective character, along with its relatively small sample size, resulted in an unavoidable selection bias. Second, the length of the research period may result in constraints. Third, there have been few studies on MR localizer sequences, so we may compare our findings. Finally, the use of MR localizer sequences and their benefits should be researched further in the future.

## Conclusions

MR localizer images form small but important part of the MR of the spine. These limited, low spatial resolution, and high field-of-view images, which are frequently overlooked, allow the radiologist to diagnose other disorders in the spine. The main purpose of this study was to recommend that radiologists closely observe MR localizer sequences because findings can be crucial for patient management, reducing patient morbidity and possibly mortality, as well as legal difficulties.
